# A comparison of force adaptation in toddlers and adults during a drawer opening task

**DOI:** 10.1038/s41598-025-87441-6

**Published:** 2025-01-29

**Authors:** Laura Faßbender, Johannes Falck, Francisco M. López, Yee Lee Shing, Jochen Triesch, Gudrun Schwarzer

**Affiliations:** 1 Department of Psychology, Faculty of Psychology and Sport Science, Justus Liebig University, Otto-Behaghel-Str. 10F, 35394 Gießen, Germany; 2 Department of Psychology, Goethe University Frankfurt am Main, Frankfurt am Main, Germany; 3 Frankfurt Institute for Advanced Studies, Frankfurt am Main, Germany; 4 Department of Physics, Goethe University Frankfurt am Main, Frankfurt am Main, Germany; 5 Center for Mind, Brain and Behavior, Universities of Marburg, Gießen, Darmstadt Germany

**Keywords:** Motor adaptation, Motor learning, Development, Cognition, Children, Physiology, Psychology

## Abstract

Adapting movements to rapidly changing conditions is fundamental for interacting with our dynamic environment. This adaptability relies on internal models that predict and evaluate sensory outcomes to adjust motor commands. Even infants anticipate object properties for efficient grasping, suggesting the use of internal models. However, how internal models are adapted in early childhood remains largely unexplored. This study investigated a naturalistic force adaptation task in 1.5-, 3-year-olds, and young adults. Participants opened a drawer with temporarily increased resistance, creating sensory prediction errors between predicted and actual drawer dynamics. After perturbation, all age groups showed lower peak speed, longer movement time, and more movement units with trial-wise changes analyzed as adaptation process. Results revealed no age differences in adapting peak speed and movement units, but 1.5- and 3-year-olds exhibited higher trial-to-trial variability and were slower in adapting their movement time, although they also adapted their movement time more strongly. Upon removal of perturbation, we found significant aftereffects across all age groups, indicating effective internal model adaptation. These results suggest that even 1.5-year-olds form internal models of force parameters and adapt them to reduce sensory prediction errors, possibly through more exploration and with more variable movement dynamics compared to adults.

**Supplementary Information:**

The online version contains supplementary material available at 10.1038/s41598-025-87441-6.

## Introduction

Around the first year of life, children use internal models to guide their grasping behavior ^1–5^. Internal models are representations of the external world used to predict and simulate behavior based on sensory information and convert them into motor actions^6,7^. Previous research indicated that infants can simulate their behavior and select appropriate actions dependent on force requirements when using both hands to lift heavy objects and a single hand for light objects^1^. Further, they lift light objects higher than heavy objects^5^ and selectively scale their forces even when no visual information is provided^4^. Moreover, they exhibit different neural responses when processing objects of varying weights^2^. However, in our dynamic environment, conditions often change unpredictably, for example when opening doors or drawers with unexpected resistances. Then, the applied force of the chosen action needs to be spontaneously adapted to allow an efficient interaction with current object’s properties. Research has shown that children aged four and older can adapt their forces^8–10^. However, it remains unclear whether and how force adaptation unfolds in even younger children. This study examines to what extent children at 1.5- and 3-years of age can adapt their forces while grasping and pulling an object whose force requirements change unpredictably. This can provide insights to which extent young children are able to adapt their internal models when experiencing mismatches between their predicted and actual sensory outcomes.

Force adaptation, a subcategory of motor adaptation, refers to a neural process within motor control whereby internal models are adapted in response to external force perturbations, such as changes in resistance when moving an object or handle^11–13^. Participants initially perform a baseline block to measure their individual motor performance. Subsequently, an adaptation block introduces an external perturbation to the resistance of the target object, resulting in sensory prediction errors, i.e. mismatches between predicted and actual sensory consequences of movements. During the adaptation block, these sensory prediction errors reduce gradually through internal model adaptation. Once the perturbation is removed, aftereffects emerge as errors in the opposite direction to the baseline, resulting from the continued use of adapted movements despite the absence of the perturbation. During a following deadaptation (or washout) block, aftereffects (over- or undershoot of movements) are gradually reduced as the internal model deadapts to the original task conditions^11–15^.

Although force adaptation in adults has been widely explored in laboratory settings^13,17–20^, and more naturalistic environments^21^, literature on the development of force adaptation in children is relatively limited. Previous studies investigated force adaptation in children aged 4–11 years^8–10^ in laboratory settings. In these studies, children performed goal-directed forearm extension and flexion movements on a robotic manipulandum that applied velocity-dependent forces altering the movement direction^8,9^, or they moved a robot handle that applied force-fields to a specific target^10^. The results demonstrated that movements were initially perturbed in all participants, regardless of age. Trial-to-trial adaptation showed age-differences only in some parameters. For instance, younger children exhibited prolonged adaptation times^8,10^ and deadaptation times^9,10^, as well as greater movement variability compared to adults^8–10^. The authors concluded that motor adaptation functions similarly across this age range, although the internal model parameters appear to be less fine-tuned and more affected by neuromotor noise in younger participants^8–10^. These findings on force adaptation align with motor adaptation studies using visuomotor adaptation tasks^16,22,23^ and gait adaptation^24–26^. However, it remains unclear when this ability starts to function and to what extent children below 4 years can adapt their forces to unpredictable changes.

**Fig. 1:**
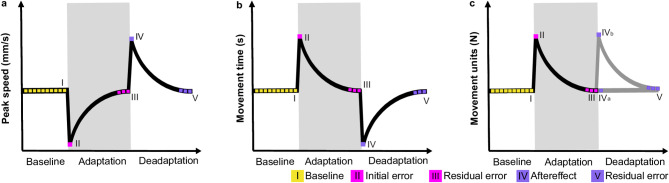
Hypothetical adaptation and deadaptation process for (a) peak speed, (b) movement time, and (c) movement units. The gray area represents adaptation trials (13–24) with increased drawer resistance. Initially, peak speed should decrease and movement time and movement units increase, signaling initial errors (II). Over time, participants should adapt, so increasing peak speed and decreasing movement time and movement units to baseline average, reflecting low residual errors (III). During the deadaptation block, we expect aftereffects characterized by overshooting peak speed and undershooting movement time (IV). No clear hypothesis for movement units was formulated because participants could either open the drawer fast and stop the drawer at the end of movement (IVa) or stop the movement directly after recognizing the low resistance and then accelerate the drawer again (IVb). By the end of deadaptation, low residual errors are anticipated if internal models were adapted (V).

In the present study, we investigated force adaptation in 1.5-year-olds who have recently acquired critical motor milestones^27–29^ and in 3-year-olds, who display nearly adult-like kinematics^3,8,29–31^. A control group of young adults was also tested. We employed an experimental, more naturalistic task than the previously reported. The approach was chosen to align with the motor and cognitive abilities of our younger age groups and to ensure that all participants had prior experience and corresponding neural representations (i.e., internal models) for the given task. In the task, participants were asked to open a drawer whose resistance was temporarily increased. Thus, participants experienced spontaneous sensory prediction errors between intended and actual drawer dynamics. Rewards were provided after each trial regardless of task performance. Consequently, sensory errors between internal models and action effects were the main drivers of the adaptation and deadaptation processes we investigated. Figure 1 presents our hypothetical adaptation and deadaptation processes of the drawer opening peak speed, movement time, and movement units. This is based on (de)adaptation curves demonstrated in previously presented studies with adults. Concerning the age comparison, we hypothesized that even 1.5- and 3-year-olds would show force adaptation and deadaptation due to their developed ability to predict and interact with objects after the first year of life^1,3,4,5,11,32–34^. However, based on force-adaptation studies with older children (≥ 4 years), we hypothesized that children would show lower adaptation and deadaptation performance, represented by a slower return of peak speed, movement time, and movement units to baseline average, along with higher residual errors at the end of both experimental blocks.

## Methods

### Participants

Thirty-seven 1.5-year-olds, twenty-five 3-year-olds, and twenty-four young adults, who served as a control group, participated in this study. The 1.5- and 3-year-olds were recruited by obtaining their birth records from local municipal councils and neighboring communities, and contacting their parents by mail or e-mail. Adult participants were undergraduate students at the Justus Liebig University Giessen. Data of eighteen 1.5-year-olds were excluded from the analysis due to lack of cooperation (*n =* 7), technical problems (*n* = 3), or early termination of the experiment (*n =* 8). Additionally, six 3-year-olds (lack of cooperation: *n =* 2, technical problems *n* = 4) and four adults (technical problems: *n* = 1, left-handedness: *n* = 3) were excluded. The final sample comprised nineteen 1.5-year-olds, nineteen 3-year-olds, and twenty young adults with a middle to high socio-economic status, and normal level of fine motor skills assessed by the Bayley-III Screening Test for fine motor skills^36^. Anthropometric data for the final sample are outlined in Table 1. All included adults were right-handed or ambidextrous (*n* = 2), as measured by the Edinburgh Handedness Inventory^37^.

The 1.5- and 3-year-olds received a gift and a certificate for participation, while adults were compensated with course credits or a 5 euro voucher. This study was conducted in accordance with the German Psychological Society (DGPs) research ethics guidelines. The Office of Research Ethics at the Justus Liebig University Giessen approved the experimental procedure and the informed consent protocol. Written informed consent was obtained from the parents of the children or the adult participants themselves before study participation.

**Table 1:** Anthropometric data of participants.

	1.5-year-olds		3-year-olds	Young adults
N	19		19	20
Age	17.4 ± 0.6 months		40.0 ± 2.2 months	22.7 ± 2.4 years
Gender (f/m/d)	7/12/0		13/6/0	18/2/0
Height (cm)	83.3 ± 2.4		100.5 ± 4.6	169 ± 8.6
Weight (kg)	11.6 ± 1.2		16.1 ± 2.1	61.3 ± 15.5
Arm length (cm)	31.4 ± 2.4		39.9 ± 2.9	66.7 ± 13.0

### Apparatus

**Fig. 2:**
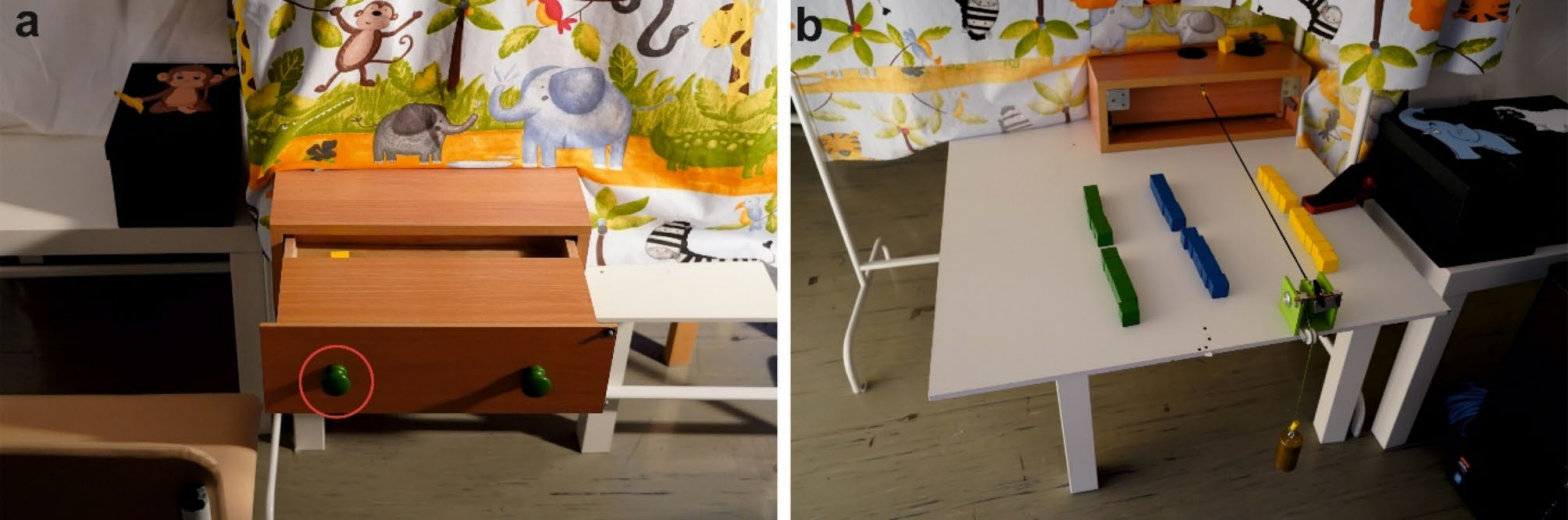
Experimental setup. (a) The fully opened drawer from the perspective of a participant. The slot was only accessible at the very end so the participants had to open the drawer completely to get cubes out of the drawer that were placed into the box cut out on the participant’s left. The red circle highlights the drawer knob that was used to open the drawer with the right hand. (b) The experimenter’s view with cubes to motivate participants and a weight to perturb the resistance of the drawer.

The experimental setup consisted of two small tables (height: 45 cm), a drawer (dimensions: 42 × 28.5 × 14 cm), an age-appropriate chair, 36 cubes (2 × 2 cm) of different colors, a box for collecting the cubes, and a curtain above the drawer (Fig. 2a). The drawer was equipped with two green knobs (scope: 10 cm, diameter: 3 cm), positioned 9 cm from each edge of the drawer. The drawer lacked roller rails, so the movement would stop immediately when no force was applied. At the back of the drawer, there was a cutout through which small cubes could be inserted into the drawer. Additionally, the drawer had an aperture (15 cm) at the upper part, requiring participants to fully open the drawer before accessing cubes through the back slot (depth: 4.5 cm). Drawer resistance manipulation was adjusted for the various age groups and varied for each age group: a weight of 150 g for 1.5-year-olds, 250 g for 3-year-olds, and 500 g for adults were used. This resistance was achieved by attaching weights via a small string that passed over a pulley located at the back of the drawer (Fig. 2b). The chosen age-related drawer resistances were piloted beforehand to ensure that the change of drawer resistance was perceived comparably across the age groups (see Supplementary Fig. S1 online).

A child-friendly curtain in front of the participants covered the experimenter and any weight adjustments or inserted cubes of the drawer. Participants sat on age-appropriate chairs (36–48 cm height) positioned to the left of the drawer. The lateral distance between the chair and the drawer knob was adjusted so that the participant’s forearm was aligned with the drawer knob. Additionally, the frontal distance between the chair and the drawer was adjusted in such a way that the drawer could be opened comfortably straight using flexion and anteversion of the right arm. In front of the participants, a box was positioned with an animal (monkey, sheep, or elephant) with a cutout (2.5 × 2.5 cm) in the cover.

Drawer kinematics were recorded using a Vicon Nexus 2.2.3 motion capture system (Vicon, Oxford, England). Six infrared cameras (Bonita) tracked the motion of two small reflective markers (6 mm in diameter) located on the front right and lateral lower edge of the drawer, with a sampling frequency of 100 Hz.

### Experimental procedure

Participants (and their parents) were welcomed at the department entrance and familiarized with the laboratory environment. Two experimenters were present during the testing for the children. One communicated with families and administered the technical equipment, while the other supervised and interacted with the child. The parents of the children and the participating students were informed about the procedure and were given consent forms to sign. Meanwhile, one experimenter engaged with the child about the animals depicted on the curtain. Once consent forms were signed, the experimental setup was explained. The experimenter opened the drawer, took the inserted cube, and handed it to the child. The participant then placed the cube into the animal box nearby. All participants were instructed to open the drawer several times to collect little cubes to “feed” the animal depicted on the box (i.e. yellow cubes as bananas for the monkey, blue cubes as water for the elephant and green cubes as grass for the sheep). Participants were asked to open the drawer using only their right hand and the left knob. The chair was positioned to the left of the drawer (see Fig. 2a), which naturally encouraged using the right hand for the task. To further guide the children, the experimenter, positioned behind the child, tapped the left knob before each trial to prompt the children to use this knob to open the drawer. Once the drawer was fully opened, the cube was collected and placed through the box’s cutout. The animal box was replaced with another one after every sixth trial to maintain participant motivation. The order of appearance of the three animals on the boxes was randomized across blocks and participants. After two warm-up trials, the experiment began with its three blocks: the baseline block (trials 3–12) with normal drawer resistance (around 50 N), the adaptation block (trials 13–24) with increased drawer resistance (adults: +500 g, 3-year-olds: +250 g, 1.5-year-olds: +150 g weight), and the deadaptation block (trials 25–36) where drawer resistance returned to baseline level by removing the weights. Participants were not informed about the change of drawer resistance before the experiment.

### Data preprocessing

Vicon Nexus 2.2.3 (Vicon, Oxford, England) was used to preprocess the kinematic data captured from the drawer. First, we reconstructed and labeled the data. Missing markers were interpolated using the pattern fill algorithm, as both markers consistently maintained the same distance and moved together in the same direction. When both markers were occluded simultaneously spline fill interpolation was applied with a maximum gap length of 500 samples^38^. A Butterworth low-pass filter with a cutoff frequency of 8 Hz was applied to the data. All following data analyses were conducted using MATLAB R2022a (MathWorks, Natick, MA, USA).

### Data analysis

We obtained the three-dimensional position of the infrared markers on the drawer for each trial and calculated drawer opening speed by numerically differentiating positional data. The movement onset was defined as the first point at which the drawer movement speed exceeded 30 mm/s. Movement offset was determined when drawer speed fell below 30 mm/s and was less than 35 mm away from the drawer’s maximum pullout position.

We calculated *peak speed* of the drawer opening as the maximum speed detected between movement onset and offset for each trial. *Movement time* defined the duration between movement onset and offset. *Movement units* indicated the number of local maxima in drawer speed and served as a measurement of motor planning. Well-planned adult movements are typically bell-shaped with exactly one movement unit, whereas infants and children often exhibit multiple *movement units*^30,39,40^.

Outliers were corrected first at the individual level. Trials deviating more than the mean ± 2.5 standard deviations from the individual’s remaining trials for peak speed [0.88%], movement time [2.70%], and movement units [1.91%] were excluded. The first adaptation and deadaptation trials were exempt from individual’s outlier correction due to their expected divergence from the remaining trials. At the group level, data (including first (de)adaptation trials) deviating more than the mean ± 2.5 standard deviations from the group mean for peak speed [0.02%], movement time [0.03%], and movement units [0.02%] were excluded.

### Statistical analyses

Statistical analyses were performed using JASP (Version 0.18.3, JASP Team 2024) and RStudio (Version 2023.12.0, RStudio Team 2023). All tests were conducted at a significance level of *α <* 0.05, with post-hoc tests Bonferroni-corrected. Errorbars and variances in statistical tests are presented as standard errors (s.e.m). For analyses in the adaptation block, repeated measures ANOVAs were conducted to test for differences in *peak speed*,* movement time*, and *movement units* between the mean of baseline block and the first adaptation trial (*initial error*), as well as between the mean of baseline and the mean of the last three adaptation trials (*residual error adaptation block*), with age as a between-subjects factor. To examine the course of adaptation, linear mixed model analyses were performed in Rstudio using the lme4 package^41^. Fixed effects included *trials* and *age*, with *subjects* treated as random effect. The significance was calculated using the lmerTest package^42^, which applies the Kenward-Rogers method for estimating degrees of freedom and generating *p*-values for mixed models. A priori defined customized contrasts were conducted when a significant fixed effect of age or the interaction of age and trials were indicated. Additionally, the trend function was used to investigate post-hoc slope differences. The model specification for the adaptation block was as follows: dependent variable ~ 1 + Trial * Age + (1|subject). Model fits and extended outputs are outlined in the Supplementary Table S1-S6 online. Similar analyses were conducted for the deadaptation process with regard to *peak speed*,* movement time*, and *movement units*. Repeated measures ANOVAs were used to investigate the aftereffect as described by the comparison of those measures between the first deadaptation trial to the baseline average, while the *residual error* in the deadaptation block compared the average of the last three deadaptation trials with the baseline average. The model specification for the linear mixed model in the deadaptation block was as follows: dependent variable ~ 1 + Trial * Age + (1|subject).

## Results

### Adaptation

**Fig. 3:**
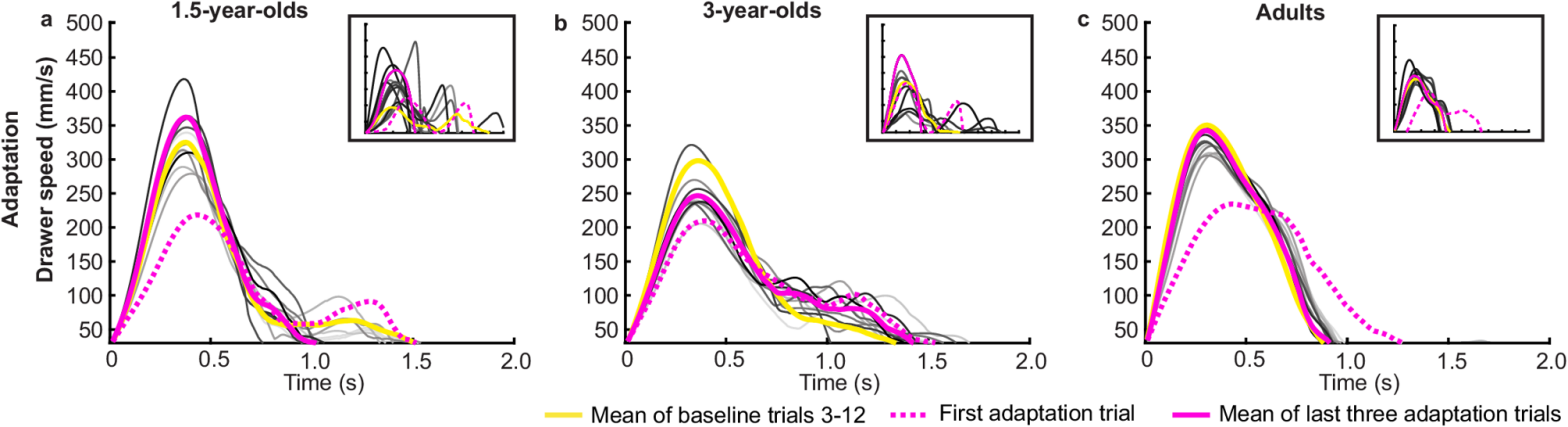
Speed profiles of drawer movement in the adaptation block. Drawer speed for averaged baseline trials 3–12 (yellow), first adaptation trial (dashed pink), averaged last three adaptation trials (solid pink), and all remaining adaptation trials (from light gray to dark gray with increasing trial number) is depicted for (a) 1.5-year-olds, (b) 3-year-olds, and (c) adults. The first adaptation trial (dashed pink) showed the slowest drawer speed and the longest movement time. By the end of the adaptation block, the solid pink line (representing the average of the last three adaptation trials) was close to the baseline average (yellow line) in each age group. Descriptively, 1.5- and 3-year-olds showed longer movement time (later intersection with x-axis) and more movement units (more local maxima) compared to adults. On the top right of each panel speed profiles of one example participant of each age group are presented.

Figure 3 shows that the speed profiles of the drawer opening movement were perturbed by the spontaneous increase in drawer resistance, but adapted in subsequent trials.

### Initial error between baseline and first adaptation trial

The 3 (age) x 2 (initial error: baseline vs. first adaptation trial) repeated measures ANOVAs for each dependent variable (peak speed, movement time, movement units) revealed significant initial errors of all variables across all age groups, indicating successful perturbation due to increased drawer resistance.

**Fig. 4:**
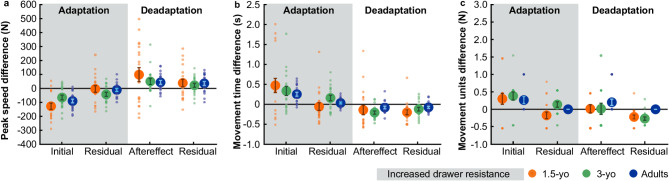
Perturbation effects for (a) peak speed, (b) movement time, and (c) movement units for 1.5-years-old (orange), 3-years-old (green), and adults (blue). Averages are depicted in circles; error bars reflect the standard errors. The adaptation block in which the drawer resistance was increased is marked in gray. The initial error is shown as a difference between the first adaptation trial and the baseline average, the residual error (adaptation) shows the difference between the average of the last three adaptation trials and the baseline average. The aftereffect indicates the difference between the first deadaptation trial and the baseline average. The residual error (deadaptation) shows the difference between the average of the last three deadaptation trials and the baseline average. For successful perturbation of drawer resistance, initial error and aftereffect should differ from baseline average. Residual errors should be close to the baseline average to indicate that the dependent variable has been fully (de-)adapted to the baseline average.

In detail, peak speed showed a main effect of initial error, *F*(1, 54) = 65.99, *p* < .001, *η*^*2*^_*p*_ = 0.55, with a significant decrease from baseline (*M* = 354.57 ± 10.19 mm/s) to the first adaptation trial (*M* = 266.12 ± 10.28 mm/s). No main effect of age, *F*(2, 54) = 1.17, *p* = .317, *η*^*2*^_*p*_ = 0.04, or interaction between age and initial error, *F*(2, 54) = 1.46, *p* = .242, *η*^*2*^_*p*_ = 0.05, was found (Fig. 4a).

Movement time revealed a significant main effect of initial error, *F*(1, 52) = 30.14, *p* < .001, *η*^*2*^_*p*_ = 0.37, with an increase from baseline (*M* = 990.89 ± 35.38 ms) to the first adaptation trial (*M* = 1338.91 ± 77.10 ms). A main effect of age was detected, *F*(2, 52) = 6.08, *p* = .004, *η*^*2*^_*p*_ = 0.19. Both, the 1.5-year-olds (*M* = 1319.41 ± 129.74 ms; *p*_*bonf*_ = .005, *d* = 0.89) and 3-year-olds (*M* = 1242.21 ± 85.66 ms; *p*_*bonf*_ = .035, *d* = 0.71) had longer movement times compared to adults (*M* = 945.28 ± 37.82 ms). There was no significant interaction between age and initial error, *F*(2, 52) = 1.06, *p* = .355, *η*^*2*^_*p*_ = 0.04 (Fig. 4b).

Movement units displayed a significant main effect of initial error, *F*(1, 54) = 18.02, *p* < .001, *η*^*2*^_*p*_ = 0.25 with an increase from baseline (*M* = 1.33 ± 0.04; *M*_*adults*_ = 1.00 ± 0.00; *M*_*3 − yo*_ = 1.46 ± 0.05, *M*_*1.5−yo*_ = 1.54 ± 0.07) to the first adaptation trial (*M* = 1.65 ± 0.08; *M*_*adults*_ = 1.26 ± 0.10; *M*_*3 − yo*_ = 1.84 ± 0.12, *M*_*1.5−yo*_ = 1.84 ± 0.16). A main effect of age was identified, *F*(2, 54) = 18.49, *p* < .001, *η*^*2*^_*p*_ = 0.41. Post-hoc tests revealed more movement units in 1.5-year-olds (*M* = 1.69 ± 0.11) compared to adults (*M* = 1.13 ± 0.10), *p*_*bonf*_ < .001, *d* = 1.33, and more movement units in 3-year-olds (*M* = 1.65 ± 0.08), *p*_*bonf*_ < .001, *d* = 1.23 compared to adults. No significant interaction between age and initial error was determined, *F*(2, 55) = 0.23, *p* = .799, *η*^*2*^_*p*_ = 0.01 (Fig. 4c). A histogram for movement units for all age groups is attached in the Supplementary Figure S2 online.

### Residual errors in adaptation block

The 3 (age) x 2 (residual error: baseline vs. mean last three adaptation trial) repeated measures ANOVAs revealed non-significant residual errors of all variables across all age groups, suggesting successful adapation.

Hence, peak speed displayed no main effect of residual error, *F*(1, 55) = 3.70, *p* = .059, *η*^*2*^_*p*_ = 0.06), indicating no significant differences between end of adaptation (*M* = 341.34 ± 11.36 mm/s) and baseline (*M* = 360.20 ± 11.49 mm/s). A main effect of age was identified, *F*(2, 55) = 7.10, *p* = .002, *η*^*2*^_*p*_ = 0.21, with higher peak speed in 1.5-year-olds (*M* = 388.22 ± 25.75 mm/s) compared to 3-year-olds (*M* = 303.10 ± 15.15 mm/s), *p*_*bonf*_ = .002, *d* = 1.06, and lower peak speed in 3-year-olds compared to adults (*M* = 360.47 ± 11.36 mm/s), *p*_*bonf*_ = .044, *d* = -0.71. The interaction between age and residual error was not significant, *F*(2, 55) = 1.43, *p* = .249, *η*^*2*^_*p*_ = 0.05 (Fig. 4a).

Movement time obtained no main effect of residual error, *F*(1, 55) = 1.21, *p* = .276, *η*^*2*^_*p*_ = 0.02, suggesting that movement time did not differ significantly between baseline (*M* = 989.64 ± 34.02 ms) and end of adaptation (*M* = 1031.58 ± 44.42 ms). A main effect of age was detected, *F*(2, 55) = 10.91, *p* < .001, *η*^*2*^_*p*_ = 0.28, with higher movement time in 1.5-year-olds (*M* = 1052.34 ± 84.54 ms) compared to adults (*M* = 831.72 ± 25.09 ms), *p*_*bonf*_ = .008, *d* = 0.82, and higher movement time in 3-year-olds (*M* = 1152.61 ± 60.24 ms) compared to adults, *p*_*bonf*_ < .001, *d* = 1.19. No significant interaction between age and residual error was observed, *F*(2, 55) = 2.32, *p* = .108, *η*^*2*^_*p*_ = 0.08 (Fig. 4b).

Movement units revealed no main effect of residual error, *F*(1, 55) = 0.09, *p* = .763, *η*^*2*^_*p*_ < 0.01. Thus, movement units did not differ significantly between baseline (*M* = 1.33 ± 0.04) and end of adaptation (*M* = 1.32 ± 0.05). We found a main effect of age, *F*(2, 55) = 34.55, *p* < .001, *η*^*2*^_*p*_ = 0.56, with more movement units in 1.5-year-olds (*M* = 1.46 ± 0.08) compared to adults (*M* = 1.00 ± 0.00), *p*_*bonf*_ < .001, *d* = 1.72, and more movement units in 3-year-olds (*M* = 1.53 ± 0.07) compared to adults, *p*_*bonf*_ < .001, *d* = 1.99. W﻿e found a significant interaction between age and residual error, *F*(2, 55) = 4.83, *p* = .012, *η*^*2*^_*p*_ = 0.15. Compared to the baseline, 1.5-year-olds had fewer movement units at the end of adaptation (*M*_*diff*_ = ˗0.17 ± 0.04), 3-year-olds had more movement units at the end of adaptation (*M*_*diff*_ = 0.14 ± 0.01), and adults had equal movement units at the end of adaptation (*M*_*diff*_ = 0.00 ± 0.00) (Fig. 4c).

### Linear mixed model trial-wise adaptation analysis

Linear mixed models were applied to each dependent variable, with trials (13–24) and age as fixed effects, and subject as a random factor, to analyze the trial-to-trial adaptation process between age groups (cf., statistical analyses). Model fits and estimates are presented in the Supplementary Tables S4-6 online.

**Fig. 5:**
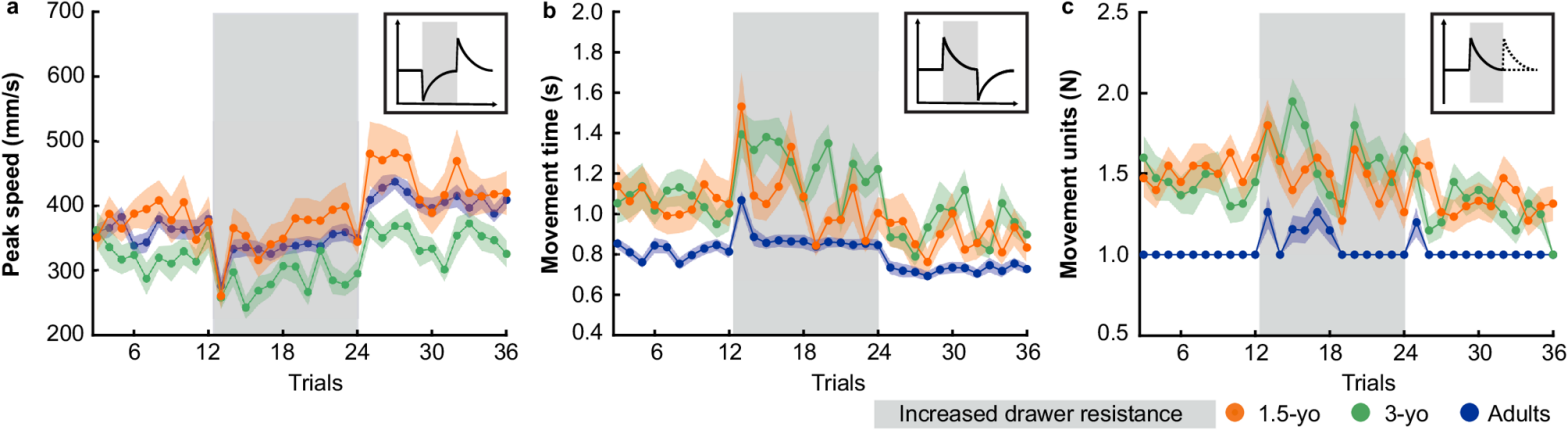
Trial-wise (de-)adaptation for (a) *peak speed*, (b) *movement time*, and (c) *movement units* averaged across 1.5-year-olds (orange), 3-year-olds (green) and adults (blue) for each trial with shaded error bars showing the standard errors. The adaptation block (trials 13–24) in which the drawer resistance was increased is marked in gray. On the top right of each panel, the theoretical behavior for each variable is illustrated.

A significant main effect of trials on peak speed was found, *F*(1, 618.29) = 29.72, *p* < .001, *η*_*p*_^*2*^ = 0.05. This supports the adaptation process (i.e. an increase) of peak speed with subsequent trials. Peak speed was not significantly different between age groups across all trials, *F*(2, 504.80) = 0.20, *p* = .822, *η*_*p*_^*2*^ < 0.01. There was no significant interaction effect between age and trials, *F*(2, 618.29) = 1.77, *p* = .171, *η*_*p*_^*2*^ < 0.01 (Fig. 5a).

We found a significant main effect of trials on movement time, *F*(1, 602.46) = 33.72, *p* < .001, *η*_*p*_^*2*^ = 0.05. Thus, movement time adapted (i.e. increased) with subsequent trials. Further, a main effect of age was identified, *F*(2, 570.34) = 7.66, *p* < .001, *η*_*p*_^*2*^ = 0.03. Customized contrasts showed that 1.5-year-olds had longer movement time compared to adults (*p* = .008), and 3-year-olds had longer movement time than adults (*p* < .001).

We found a significant interaction between age and trials, *F*(2, 602.46) = 3.30, *p* = .038, *η*_*p*_^*2*^ = 0.03. Post-hoc analysis with customized contrasts and trend function revealed that 1.5-year-olds had the greatest reduction in movement time across trials with an estimated slope of ˗33.6 ms per trial, followed by 3-year-olds showing a significant reduction in movement time of ˗21.2 ms per trial, and adults exhibiting a movement time reduction of ˗10.1 ms per trial. Statistically 1.5-year-olds reduced their movement time significantly more than adults (*p* = .028). Descriptively, children needed more trials to adapt their movement time and had a higher trial-to-trial variability than adults that reduced their movement time to baseline level already in the second trial (see Fig. 5b). To check the trial-by-trial variability statistically we conducted a 1 × 3(age) ANOVA on the participants individual standard deviation of the movement time divided by the individual mean of movement time^10^. A significant main effect of age on trial-to-trial variability was found, *F*(2, 55) = 15.48, *p* < .001, *η*_*p*_^*2*^ = 0.36. Post-hoc tests revealed a significantly higher trial-to-trial variability in 1.5-year-olds (*M* = 0.39 ± 0.05 ms) compared to adults (*M* = 0.13 ± 0.02 ms), *p*_*bonf*_ < .001, *d* = 1.73, and higher trial-to-trial variability in 3-year-olds (*M* = 0.31 ± 0.03 ms) compared to adults, *p*_*bonf*_ < .001, *d* = 1.23.

For movement units, we found a main effect of trials, *F*(1, 610.83) = 20.85, *p* < .001, *η*_*p*_^*2*^ = 0.03. Movement units were reduced with subsequent trials. A main effect of age, *F*(2, 663.56) = 3.79, *p* = .023, *η*_*p*_^*2*^ = 0.01 was found, with 1.5-year-olds having more movement units compared to adults (*p* < .001), as well as 3-year-olds having more movement units compared to 3-year-olds (*p* < .001). No interaction between age and trials was detected, *F*(2, 610.82) = 0.21, *p* = .813, *η*_*p*_^*2*^ < 0.01 (Fig. 5c).

### Deadaptation

**Fig. 6:**
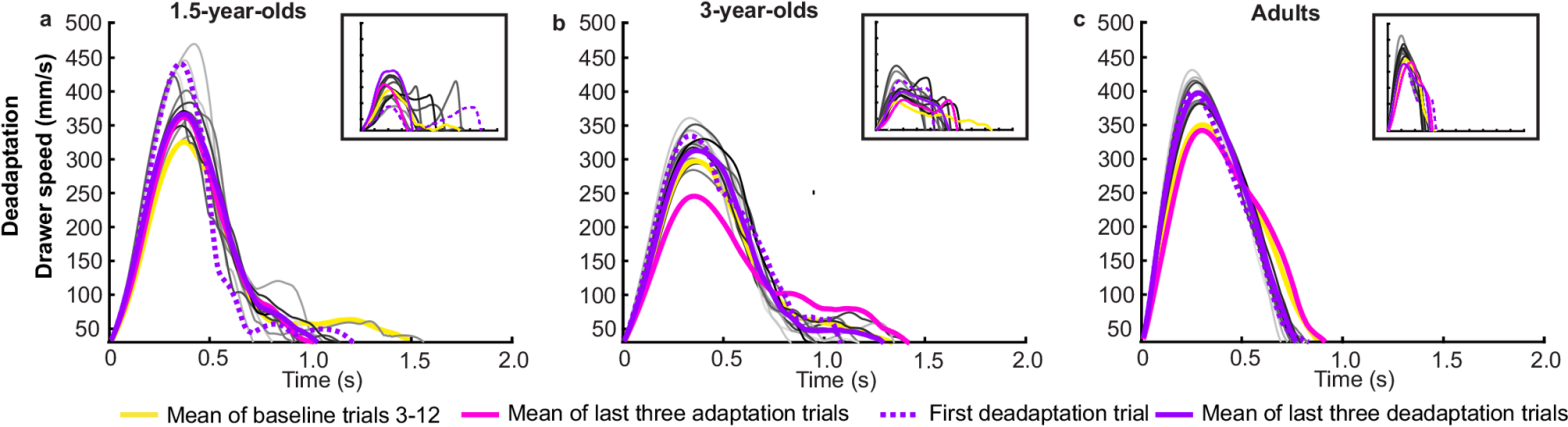
Speed profiles of drawer movement in the deadaptation block. Drawer speed for the averaged baseline trials (yellow), averaged last three adaptation trials (pink), first deadaptation trial (dotted purple) and averaged last three deadaptation trials (solid purple), and all remaining trials (from light gray to dark gray) for (a) 1.5-year-olds, (b) 3-year-olds, and (c) adults. At the beginning of the deadaptation block, the drawer speed overshot the last adaptation trial value (dotted purple line) in all three age groups. In tendency, this overshoot decreased with ongoing trials (solid purple line) in the direction to the baseline level (yellow line) and the last adaptation trials (pink line) in each age group. However, it seems that all age groups did not fully deadapt the drawer speed profiles to the level of the previous experimental blocks. Also, in the deadaptation block, descriptively, adults had fewer movement units and shorter movement time than children. On the top right of each panel speed profiles of one example participant of each age group are presented.

Figure 6 presents the averaged speed profiles of the drawer movement within the 12 trials of the deadaptation block compared to the averaged baseline trials and the averaged last three adaptation trials for all three age groups.

### Aftereffects between first deadaptation trial and baseline

To investigate whether aftereffects of the adaptation block existed when reducing the drawer resistance back to baseline level, we conducted for each dependent variable a 3 (age) x 2 (aftereffect: baseline vs. first deadaptation trial) repeated measures ANOVA.

Peak speed showed a significant main effect of aftereffects, *F*(1, 53) = 10.09, *p* = .002, *η*^*2*^_*p*_ = 0.16. Thus, peak speed in the first trial of deadaptation (*M* = 424.97 ± 19.84 mm/s) overshot peak speed of the baseline (*M* = 358.57 ± 11.84 mm/s) significantly. A main effect of age was observed, *F*(2, 53) = 5.12, *p* = .009, *η*^*2*^_*p*_ = 0.16, with 1.5-year-olds (*M* = 439.40 ± 38.96 mm/s) having higher peak speed than 3-year-olds (*M* = 346.96 ± 18.56 mm/s), *p*_*bonf*_ = .007, *d* = 0.79. No significant interaction between age and aftereffect was revealed, *F*(2, 53) = 0.63, *p* = .537, *η*^*2*^_*p*_ = 0.02 (Fig. 4a).

Movement time exhibited a main effect of aftereffects, *F*(1, 52) = 5.32, *p* = .025, *η*^*2*^_*p*_ = 0.09. Movement time at the first deadaptation trial (*M* = 844.73 ± 44.60 ms) undershot the baseline average (*M* = 971.80 ± 33.43 ms). A main effect of age was shown, *F*(2, 52) = 7.56, *p* = .001, *η*^*2*^_*p*_ = 0.23, indicating that 1.5-year-olds (*M* = 1000.35 ± 97.98 ms) had longer movement time than adults (*M* = 774.30 ± 30.26 ms), *p*_*bonf*_ = .002, *d* = 0.81, and 3-year-olds (*M* = 957.58 ± 44.45 ms) had higher movement time compared to adults, *p*_*bonf*_ = .014, *d* = 0.66. No significant interaction between age and aftereffect was detected, *F*(2, 52) = 0.31, *p* = .734, *η*^*2*^_*p*_ = 0.01 (Fig. 4b).

Movement units did not reveal a main effect of aftereffect, *F*(1, 54) = 1.14, *p* = .290, *η*^*2*^_*p*_ = 0.02. However, a main effect of age was present, *F*(2, 54) = 11.49, *p* < .001, *η*^*2*^_*p*_ = 0.30, with more movement units in 1.5-year-olds (*M* = 1.54 ± 0.10) compared to adults (*M* = 1.10 ± 0.09), *p*_*bonf*_ < .001, *d* = 1.06 and more movement units in 3-year-olds (*M* = 1.47 ± 0.11) compared to adults, *p*_*bonf*_ = .001, *d* = 0.88. There was no significant interaction between age and aftereffect, *F*(2, 54) = 0.65, *p* = .527, *η*^*2*^_*p*_ = 0.02 (Fig. 4c).

### Residual error in deadaptation block

To investigate whether participants deadapted their movements to the baseline level at the end of deadaptation, we used again 3 (age) x 2 (residual error: mean of last three deadaptation trials vs. baseline) repeated measures ANOVAs for each dependent variable.

Peak speed showed a main effect of residual error, *F*(1, 55) = 10.45, *p* = .002, *η*^*2*^_*p*_ = 0.16, with still higher peak speed at the end of deadaptation (*M* = 392.71 ± 11.88 mm/s) compared to baseline (*M* = 360.20 ± 11.49 mm/s). A significant main effect of age was shown, *F*(2, 55) = 4.67, *p* = .013, *η*^*2*^_*p*_ = 0.15, with higher peak speed in 1.5-year-olds (*M* = 409.56 ± 27.29 mm/s) compared to 3-year-olds (*M* = 335.54 ± 14.91 mm/s), *p*_*bonf*_ = .012, *d* = 0.87. No significant interaction was revealed between age and residual error, *F*(2, 55) = 0.25, *p* = .780, *η*^*2*^_*p*_ < 0.01 (Fig. 4a).

For movement time, we found a significant main effect of residual error, *F*(1, 55) = 17.61, *p* < .001, *η*^*2*^_*p*_ = 0.24, with still higher movement time at the end of deadaptation (*M* = 986.64 ± 34.02 ms) compared to baseline (*M* = 855.32 ± 28.83 ms). A main effect of age, *F*(2, 55) = 9.69, *p* < .001, *η*^*2*^_*p*_ = 0.26, showed that 1.5-year-olds had longer movement time (*M* = 981.47 ± 67.08 ms) than adults (*M* = 777.18 ± 25.82 ms), *p*_*bonf*_ = .003, *d* = 0.94, and 3-year-olds (*M* = 1011.86 ± 48.54 ms) had longer movement time than adults, *p*_*bonf*_ < .001, *d* = 1.08. The interaction between age and residual error was not significant, *F*(2, 55) = 1.33, *p* = .272, *η*^*2*^_*p*_ = 0.05 (Fig. 4b).

Movement units displayed a significant main effect of residual error, *F*(1, 55) = 24.20, *p* < .001, *η*^*2*^_*p*_ = 0.31, with fewer movement units at the end of deadaptation (*M* = 1.17 ± 0.03) compared to baseline (*M* = 1.33 ± 0.04). A main effect of age was determined, *F*(2, 55) = 40.34, *p* < .001, *η*^*2*^_*p*_ = 0.60, with more movement units in 1.5-year-olds (*M* = 1.43 ± 0.06) compared to adults (*M* = 1.00 ± 0.00), *p*_*bonf*_ < .001, *d* = 2.16, and 3-year-olds (*M* = 1.33 ± 0.05) having more movement units than adults, *p*_*bonf*_ < .001, *d* = 1.62. The interaction between age and residual error was significant, *F*(2, 55) = 6.34, *p* = .003, *η*^*2*^_*p*_ = 0.19. 1.5-year-olds had fewer movement units at the end of adaptation compared to baseline, while 3-year-olds had slightly more movement units, and adults had again only a single movement unit as in the baseline (*M*_*diff_1.5−yo*_ = ˗0.22 ± 1.43; *M*_*diff_3−yo*_ = 0.27 ± 1.33, *M*_*diff_adults*_ = 0.00 ± 0.00) (Fig. 4c).

### Linear mixed model trial-wise deadaptation analysis

Linear mixed models were applied to each dependent variable, with trials (25–36) and age as fixed effects, and subject as a random factor.

For peak speed, we found a significant main effect of trials, *F*(1, 613.52) = 12.27, *p* < .001, *η*_*p*_^*2*^ = 0.02, indicating that peak speed was significantly deadapted. No significant main effect of age, *F*(2, 653.55) = 3.01, *p* = .050, *η*_*p*_^*2*^ < 0.01 was detected. The interaction between age and trials, *F*(2, 613.52) = 1.22, *p =* .295, *η*_*p*_^*2*^ < 0.01 was not significant. This suggests that peak speed was not reduced differently with subsequent trials between age groups (Fig. 5a).

For movement time, we found no main effect of trials, *F*(1, 599.77) = 0.23, *p* = .634, *η*_*p*_^*2*^ < 0.01. This suggests that movement time was not deadapted. We also found no main effect of age, *F*(2, 644.76) = 0.90, *p =* .408, *η*_*p*_^*2*^ < 0.01. Furthermore, the interaction between trials and age was not significant, *F*(2, 599.74) = 0.75, *p* = .475, *η*_*p*_^*2*^ < 0.01 (Fig. 5b).

However, a main effect of trials on the number of movement units, *F*(1, 616.97) = 9.49, *p =* .002, *η*_*p*_^*2*^ = 0.02 was found. Thus, movement units decreased with subsequent trials. No main effect of age, *F*(1, 651.05) = 2.06, *p =* .128, *η*_*p*_^*2*^ < 0.01 was revealed as well as and no interaction between age and trials, *F*(2, 616.96) = 0.49, *p =* .610, *η*_*p*_^*2*^ < 0.01 (Fig. 5c).

## Discussion

This study explored how 1.5- and 3-year-olds adapt their internal models compared to adults using a naturalistic force adaptation task. Participants opened a drawer with varying resistance across three blocks: baseline (normal resistance around 50 N), adaptation (increased resistance), and deadaptation (baseline resistance). To our knowledge, this is the first study focusing on force adaptation in children under 4 years. This approach isolated the sensory error between predicted and actual action effects as primary driver of adaptation and deadaptation processes.

### Force adaptation behavior across all age groups

The unexpected increase of drawer resistance significantly perturbed the drawer opening in all age groups, indicated by lower peak speed, longer movement time and more movement units. No differences in initial errors across age groups suggest that the adjusted weights (500 g for adults, 250 g for children and 150 g for infants) successfully and comparably perturbed drawer dynamics (cf. Supplementary Material).

1.5- and 3-year-olds exhibited trial-to-trial force adaptation that was not different to adults in peak speed and movement units. Peak speed increased while movement units decreased within subsequent adaptation trials. This extends existing literature that also children as young as 1.5 years show force adaptation comparable to adults^8–10^. Further, it supports evidence from weight perception studies^1–5^ that internal models of everyday movement help to select appropriate action and facilitate adapting actions to unpredictable changes. Moreover, it aligns with motor adaptation studies on walking^24–26^ and sitting^35^ before four years of age.

Despite similarities in adaptation, there were age differences in movement time. Adults adapted their movement time quickly within the first adaptation trials, whereas 1.5- and 3-year-olds needed longer but therefore showed stronger adaptation processes, possibly due to their higher movement time at the beginning of the experiment and adaptation block. Further, children’s trial-to-trial variability was higher compared to adults. This supports other findings that children (> 4 years) show more variable and slower trial-to-trial adaptation than adults^8–10^, potentially due to less fine-tuned internal models^9^ and ongoing nervous system development^10^. This result highlights that variability appears to be an important component of motor learning and development^28,43^. Indeed, motor development is characterized by variability in trajectory, velocity, amplitude, and duration of movement, typically including multiple submovements that can be indicated by more movement units directed toward the object and an indicator for the typical development of motor abilities^28,35,40^. In our study, children were likely still using a more explorative control policy to strengthen their motor skills, which might have resulted in higher trial-to-trial variability, especially in 1.5-year-olds. Additionally, adults showed higher peak speed, shorter movement time and fewer movement units than children, indicating that children’s motor performance still differed from adult levels. This is in line with the understanding that motor control fine-tuning continues to develop through childhood, influenced by ongoing changes in the corticospinal tract^44,45^.

At the end of adaptation, across all age groups peak speed and movement time returned to baseline, indicating successful adaptation. In contrast, adaptation in terms of the number of movement units varied across age groups. After adaptation, 1.5-year-olds used fewer movement units (*M*_*1.5-yo*_ = 1.37 ± 0.09) compared to baseline (*M*_*1.5-yo*_ = 1.54 ± 0.07), while 3-year-olds required more movement units (*M*_*3-yo*_ = 1.60 ± 0.08) compared to baseline (*M*_*3-yo*_ = 1.46 ± 0.05). We assume that the lower number of movement units observed in the 1.5-year-olds after adaptation is due to the fact that many of them required multiple movement units even during the baseline trials, reduced them in the course of trials which is why they showed a reduction even during adaptation. Adults were able to complete the movement using a single movement unit after adaptation, as well as during the baseline (one movement unit – floor effect). Movement units indicate motor planning quality, with fewer units suggesting advanced forward planning and more movement units suggesting reliance on online control mechanisms^30,39,40^. In our drawer-opening task, using one or multiple movement units could be interpreted differently. Ideally, one movement unit with a soft stop shows optimal motor planning. However, one unit can also result from excessive force with a harsh stop, which is not necessarily more advanced than pulling, pausing, and continuing gently, leading to multiple units. For instance, 1.5-year-olds might used one unit with a harsh stop, while 3-year-olds used more units with a soft stop. Therefore, fewer movement units do not always indicate better motor planning in our task. Thus, we consider movement units a weaker indicator of motor planning quality compared to peak speed and movement time.

### Force deadaptation across all age groups

All age groups showed aftereffects in peak speed and movement time when drawer resistance returned to baseline, indicating successful internal model adaptation. In response to the aftereffect, all age groups deadapted their peak speed and movement units to a comparable extent with subsequent trials. However, by the end of deadaptation, residual errors in peak speed, movement time, and movement units remained different from baseline across all age groups. Peak speed was still higher by the end of deadaptation, while movement time was still shorter than in the baseline, indicating incomplete deadaptation. While previous studies suggested 10–12 trials per block were sufficient for adaptation and deadaptation^8,9^, more trials might be necessary for a complete deadaptation of all parameters (50 trials/block^10^, 40 trials/ block^16^). The number of movement units was lower than in the baseline. This reduction may be attributed to improved motor planning with repetitions^39^.

### Limitations and future implications

The current study has some limitations that should be considered in future research and when interpreting the results. The drop-out rate was relatively high, especially among 1.5-year-olds who tend to have higher drop-out rates due to lack of cooperation (e.g., distraction by technical equipment, behind-the-scene curiosity or movement restlessness). The number of trials was relatively low due to the methodological adjustments necessary for testing the young age groups. Thus, we cannot conclude whether the deadaptation process was not completed due to the low number of trials or for other reasons. Unlike other force adaptation tasks^8–10^, we did not provide a specific task goal or outcome feedback. Instead, we used the individual baseline average as individual adaptation goal and relied solely on sensor prediction errors to drive adaptation Thus, our task was more naturalistic and individualized than others and we could show that children can adapt their force exclusively through sensory (prediction) error. However, in further studies, a specific task goal with the possibility of task errors may be included to investigate how young children adapt in more restricted circumstances^46^. Further studies may also consider the muscle pre-activation (e.g., measured with EMG) to obtain data about the predicted force application and the correction of muscle activation during the movement.

In summary, this study provides evidence that 1.5- and 3- year-olds applied force adaptation to reduce sensory prediction errors, thereby matching their expected and actual movement outcomes. This suggests that internal models are built and adapted at a very young age when the environment changes unpredictably, making this process a very early component of motor control. To understand whether this is an even earlier skill, we look forward to future research that further reduces the age of study and examines force adaptation in infants during the first year of life.

## Electronic supplementary material

Below is the link to the electronic supplementary material.

Supplementary Material 1

## Data Availability

Data and analysis code related to this study can be publicly found at: DOI 10.17605/OSF.IO/B4H9V.
