# Supplementary material for: A comparison of force adaptation in toddlers and adults during a drawer opening task
Source: Sci Rep. 2025 Jan 29;15:3699. doi: 10.1038/s41598-025-87441-6 (PMC11779916; doi:10.1038/s41598-025-87441-6)
Supplement: Supplementary file 1 — Supplementary Material 1 [file 41598_2025_87441_MOESM1_ESM.pdf]

Supplementary Material

A. Piloting of different drawer resistances

To account for age-differences in maximum strength, we adapted the weights to each age group. In a piloting on three adults, six 3-year-olds and three 1.5-year-olds, we tested different weights (50 g, 100 g, 150 g, 200 g, […], 450 g, 500 g). Adults rated the opening of the drawer with an additional 500 g as a recognizable perturbation, but still as a possible drawer resistance from everyday life. When using a weight of 250 g in 3-year-olds, the drawer opening movement was comparably perturbed to adults and the children recognized that verbally as well. With this weight, it was still possible for them to open the drawer several times without fatigue. Similar observations were recognized in 1-5-year-olds using an additional weight of 150 g. Thus, the weights were set in this specific (age-adapted) way (adults: 500 g, 3-year-olds: 250 g, 1.5-year-olds: 150 g). The results show that the selected weights perturbed the peak speed and the movement time without any significant difference (cf., results). The perturbation effect (i.e., initial error defined as the difference between the first trial of the adaptation block (with increased drawer resistance) and the baseline average) induced a decreased peak speed in all age groups (M_{adults} = -90.12 ± 12.27 mm/s; $M_{\text{3-year-olds}}$ = -67.53 ± 16.32 mm/s; $M_{\text{1.5-year-olds}}$ = - 109.30 ± 23.49 mm/s) and an increased movement time (M_{adults} = 245.65 ± 40.99 ms, $M_{\text{3-year-olds}}$ = 349.90 ± 14.66 ms, $M_{\text{1.5-year-olds}}$ = 470.07 ± 158.91 ms).

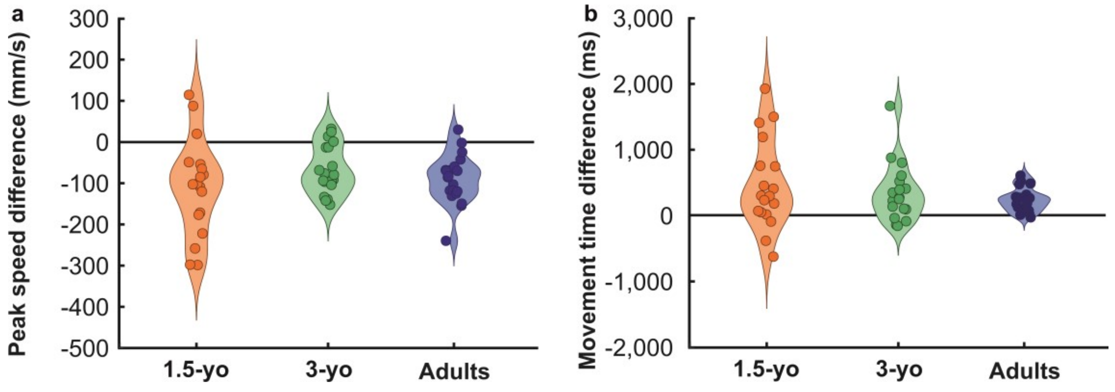

Figure S1. Perturbation effect of increased drawer resistance for (a) peak speed and (b) movement time. The perturbation effect was calculated as the differences between the first adaptation trial and the mean of the baseline block for 1.5-year-olds (orange), 3-year-olds (green), and adults (blue).

B. Movement units

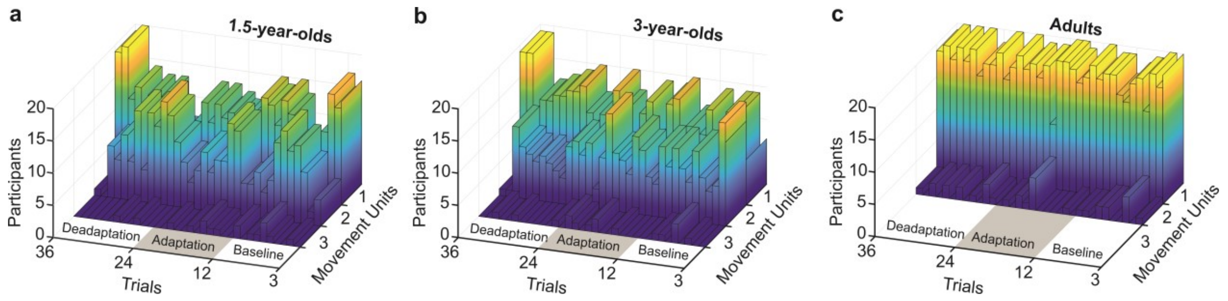

Figure S2. Histogram on movement units for (a) 1.5-year-olds, (b) 3-year-olds, and (c) 1.5-year-olds. It is shown that adults had either one or two movement units, while 3-year-olds and 1.5-year-olds had more often also two or three movement units. This shows a less perfectly forward planning of their opening movement.

33 C. Linear mixed model outputs for adaptation

Table S1. LMM output of the adaptation block and the variable peak speed. The estimates and model fits of the linear mixed model are presented.

**Term**	*Estimate*	*SE*	*CI*	*t*	*p*
Intercept	256.53	30.70	196.26 - 316.80	8.36	**<0.001**
Trial	4.31	1.44	1.48 - 7.15	2.99	**0.003**
3-year-olds	-26.27	43.95	-112.56 - 60.03	- 0.60	0.550
1.5-year-olds	-20.15	44.20	- 106.94 - 66.64	- 0.46	0.649
Trial x 3-yo	-1.47	2.070	- 5.53 - 2.59	- 0.71	0.478
Trial x 1.5-yo	2.47	2.08	- 1.62 - 6.56	1.19	0.236
AIC	7928.41	σ^2	5885.84		
BIC	7964.58	Marginal R^2 /	0.12/		
		Conditional R^2	0.48		

Table S2. LMM output of the adaptation block and the variable movement time. The estimates and model fits of the linear mixed model are presented

**Term**	*Estimate*	*SE*	*CI*	*t*	*p*
Intercept	1060.85	128.953	807.64-1314.06	8.23	**<0.001**
Trial	-10.14	6.25	-22.41-2.14	- 1.62	0.105
3-year-olds	-593.55	185.44	229.43-957.67	3.20	**0.001**
1.5-year-olds	657.27	187.18	289.73-1024.80	3.51	**<0.001**
Trial x 3-yo	-11.07	9.00	-28.73-6.60	-1.23	0.219
Trial x 1.5-yo	-23.43	9.12	-41.33 - -5.53	-2.57	**0.010**
AIC	9650.61	σ^2	109776.53		
BIC	9686.58	Marginal R^2 /	0.17/		
		Conditional R^2	0.45		

Table S3. LMM output of the adaptation block and the variable movement units. The estimates and model fits of the linear mixed model are presented

**Term**	*Estimate*	*SE*	*CI*	*t*	*p*
Intercept	1.46	0.17	1.13-1.80	8.57	**<0.001**
Trial	-0.02	0.01	-0.04- -0.00	-2.30	**0.022**
3-year-olds	0.58	0.24	0.11 - 1.05	2.43	**0.015**
1.5-year-olds	0.56	0.24	0.09 -1.04	2.34	**0.020**
Trial x 3-yo	-0.00	0.01	-0.03-0.02	-0.09	0.927
Trial x 1.5-yo	-0.01	0.01	-0.03-0.02	-0.60	0.551
AIC	941.28	σ^2	0.20		
BIC	977.34	Marginal R^2 /	0.21/		
		Conditional R^2	0.32		

D. Linear mixed model outputs for deadaptation

Table S4. LMM output of the deadaptation block and the variable peak speed. The estimates and model fits of the linear mixed model are presented

**Term**	*Estimate*	*SE*	*CI*	*t*	*p*
**Intercept**	470.80	55.74	361.35-580.25	8.45	**<0.001**
Trial	-1.99	1.72	-5.36-1.37	-1.17	0.244
3-year-olds	-34.12	79.84	-190.88-122.64	-0.43	0.669
1.5-year-olds	150.94	79.56	-5.28 - 307.16	1.90	0.058
Trial x 3-yo	-0.88	2.45	-5.69 - 3.93	-0.36	0.721
Trial x 1.5-yo	-3.68	2.45	-8.48 - 1.13	-1.50	0.133
AIC	8090.54	σ^2	8049.48		
BIC	8126.65	Marginal R^2 /	0.110/		
		Conditional R^2	0.521		

Table S5. LMM output of the deadaptation block and the variable movement time. The estimates and model fits of the linear mixed model are presented

Term	*Estimate*	*SE*	*CI*	*t*	*p*
Intercept	672.06	173.963	330.47 – 1013.66	3.863	**<0.001**
Trial	1.83	5.549	-9.06 – 12.73	0.330	0.741
3-year-olds	83.64	251.218	-409.65 – 576.94	0.333	0.739
1.5-year-olds	329.08	253.241	-168.18 – 826.35	1.299	0.194
Trial x 3-yo	4.66	8.018	-11.08 – 20.41	0.581	0.561
Trial x 1.5-yo	-5.42	8.091	-21.31 – 10.47	-0.670	0.503
AIC	9407.73	σ^2	86058.41		
BIC	9443.66	Marginal R^2 /	0.080/		
		Conditional R^2	0.283		

Table S6. LMM output of the deadaptation block and the variable movement units. The estimates and model fits of the linear mixed model are presented

**Term**	*Estimate*	*SE*	*CI*	*t*	*p*
Intercept	1.25	0.22	0.82 - 1.69	5.628	**<0.001**
Trial	-0.01	0.01	–0.02 - 0.01	–1.082	0.280
3-year-olds	0.59	0.32	–0.05 - 1.22	1.823	0.069
1.5-year-olds	0.53	0.32	–0.10 - 1.17	1.662	0.097
Trial x 3-yo	-0.01	0.01	–0.03 - 0.01	-0.988	0.323
Trial x 1.5-yo	-0.01	0.01	–0.03 - 0.01	-0.563	0.574
AIC	722.95	σ^2	0.15		
BIC	759.09	Marginal R^2 /	0.134/		
		Conditional R^2	0.243		
